# Concrete-Filled Prefabricated Cementitious Composite Tube (CFPCCT) under Axial Compression: Effect of Tube Wall Thickness

**DOI:** 10.3390/ma15228119

**Published:** 2022-11-16

**Authors:** Bi Kai, A. B. M. A. Kaish, Norhaiza Nordin

**Affiliations:** 1 Department of Civil Engineering, Infrastructure University Kuala Lumpur, Kajang 43000, Malaysia; 2 Department of Civil Engineering, Universiti Kebangsaan Malaysia, Bangi 43600, Malaysia

**Keywords:** prefabricated construction, concrete-filled column, cementitious composite tube reinforcement, mechanical property, structure behavior

## Abstract

Research on different prefabricated cementitious composites for constructing composite concrete columns is comparatively more limited than that of concrete filled steel tube columns. The main objective of this study was to observe the axial compressive behavior of concrete-filled prefabricated cementitious composite tube (CFPCCT) specimens. In the CFPCCT composite column, the spiral steel bar is arranged as a hoop reinforcement in the cementitious tube before its prefabrication. Following this, the concrete is poured into the prefabricated cementitious composite tube. The tube is able to provide lateral confinement and can carry the axial load, which is attributed to the strength of CFPCCT composite column. The effect of tube wall thickness on the behavior of CFPCCT is studied in this research. A total of eight short-scale CFPCCT composite columns, with three different tube wall thicknesses (25 mm, 30 mm and 35 mm), are tested under axial compressive load. The cementitious composite tube-confined specimens showed a 24.7% increment in load-carrying capacity compared to unconfined specimens. Increasing the wall-thickness had a positive impact on the strength and ductility properties of the composite column. However, poor failure behavior was observed for thicker tube wall. Therefore, concrete-filled cementitious composite tube columns can be considered as an alternative and effective way to construct prefabricated concrete columns.

## 1. Introduction

Concrete has excellent characteristics, such as compressive strength, durability, easy modification, and can be poured in a wide range of shapes. As a load-carrying component of structures, concrete columns play a crucial role in both industrial and civil buildings [1,2]. However, the main shortcomings of concrete materials are their poor deformability and low tensile strength [2]. Therefore, rather than using plain concrete, the cement based composite material would be widely used in future. It is expected that cement-based composites have higher strength, greater elastic modulus and better deformability [3,4]. Due to the birth of superplasticizer, the production and application of high-strength fiber reinforced cementitious composites became possible [5].

The construction component of cylindrical columns today has multiple solutions for structural performance. However, a large number of studies have concentrated on material performance enhancement, adding fiber reinforcement and hoop confinements to achieve better performance [4]. Cementitious composites are one of the most widely used building materials for the construction of infrastructure. Kaish et al. studied cementitious composite ferrocement jacketed circular and square concrete columns under axial compression [6,7,8]. Later, Kaish et al. proposed L and U shaped prefabricated ferrocement jacket for strengthening square concrete column [9]. Lim et al. studied concrete-filled hollow PC columns and reported the structural performance of initial stiffness, maximum strength and displacement ductility under seismic loading [10]. Hosoya and Asano studied precast concrete shell columns and reported a cracking pattern and failure behavior [11]. Liang-jin et al. studied concrete-filled engineered cementitious composite (ECC) tubular piers and presented the first cracking stress, ultimate loading and ultimate stress [12]. Pan et al. [13] investigated the seismic behavior of plain concrete filled ECC formwork. They have observed the effect of the slenderness ratio of such composite concrete columns. Meng and Khayat [14] investigated RC columns cast in stay-in-place prefabricated ultra-high-performance concrete (UHPC) formwork. They have presented an experimental and numerical study using the ABAQUS finite element package. Xiao and Ma [15] conducted the seismic retrofitting of RC circular columns with inadequate lap length using prefabricated fiber reinforced concrete (FRC) jackets. Shao et al. [16] proposed two different techniques for improving the seismic performance of shear-efficient RC columns, without increasing the cross-section of the column applicable, for mid-rise to high-rise buildings using UHPC jacketing. Hung et al. [17] conducted research on innovative UHPC jacketing solutions for shear-deficient reinforced concrete columns. These solutions included cast-in-place UHPC jackets, as well as prefabricated UHPC panels. Guan et al. [18] recommended for the plastic hinge region of precast columns to be strengthened with locally produced UHPC jackets. Tian et al. [19] reported numerical and experimental studies on the tubular column made of concrete filled ultra-high-performance concrete. Zhu et al. [20] and Zhang et al. [21] investigated the long-term creep and shrinkage behavior of concrete filled UHPC tubular columns under axial compression. They also developed numerical models to determine the creep and shrinkage coefficient. Shan et al. [22] investigated the seismic capacity of concrete filled cement-based tubular composite column systems. Their results confirm that this type of column carries significantly higher lateral load and absorbs higher seismic energy compared to the conventional columns.

All of these studies show the high potential for field application of prefabricated cementitious composite tubes for composite concrete columns. It combines the feasible characteristics of cementitious composites and the versatility of prefabrication, thus creating a high-performance feasible product. However, a limited number of research has been reported in the area of cementitious composites as a prefabricated confining material. Tube thickness is an important parameter that needs to be investigated in detail. Previously reported research did not study this aspect for normal strength cementitious composite tube composite columns. Therefore, this study investigated the tube thickness effect on the behavior of concrete filled prefabricated normal strength cementitious composite tubes. Three different tube thicknesses were investigated in this study under axial compression. The following sections reports the methodologies followed and discusses the obtained results in detail.

## 2. Experimental Program

**Figure 1:**
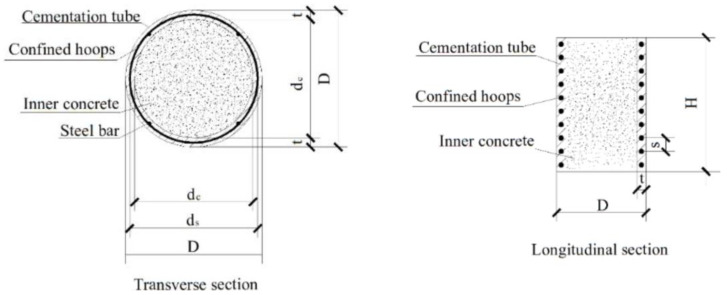
Transverse and longitudinal section of specimens.

For the specimen series, a total of 8 short-scale columns are tested under an axial compression load. All specimens were 300 mm in total height and 210 mm in overall diameter. The main testing parameter was the wall thickness of the composite tube. The spiral reinforcement was positioned closely, at the middle point of the tube. The spiral spacing was selected as constant for all of the specimens, which was 20 mm. The wall thickness varied among 25 mm, 30 mm and 35 mm, respectively, as the main variable in this study. These thicknesses were chosen based on practical considerations. Placing the hoop reinforcement is difficult in the cementitious composite tubes where less than 25 mm thickness was chosen. On the other hand, a thickness above 25 mm is impractical for small size specimens. Sectional details of tested specimens are shown in Figure 1. All other specimen details are shown in Table 1.

### 2.1. Material

#### 2.1.1. Cement

The binding materials used to prepare the concrete and the cementitious composite tube specimens were locally sourced ordinary Portland cement. The strength grade was 42.5 N ordinary Portland cement.

#### 2.1.2. Fine & Coarse Aggregates

The coarse aggregate used in concrete was downgraded crushed gravel with a maximum diameter of 12.5 mm. The locally available fine river sand (percentage passing 600 μm sieve approximate 40%) was used for both the core concrete and tube fabrication.

#### 2.1.3. Confining Hoops

**Figure 2:**
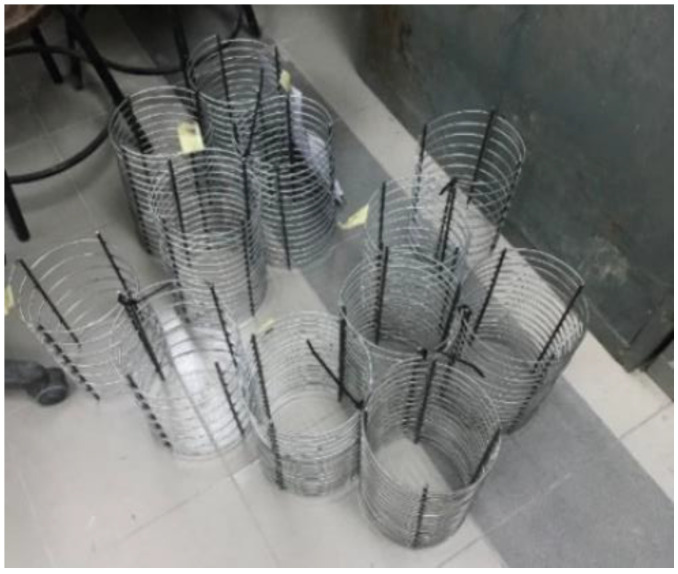
Confining hoop reinforcements.

The reinforcement (Figure 2) chosen for the spiral hoops was a 3.5 mm diameter steel bar with 350 MPa yield strength. Hoop spacing is provided in Table 1.

#### 2.1.4. Composite Materials

Ordinary Portland cement and silica fume (specific surface area of 22,500 m^2^/kg) were used as a binder in the composite mix. Quartz sand, with a maximum size 0.4 mm, was mixed with the binder, with a ratio of 1:1. Corrugated steel-fibers, with a 0.5 mm nominal diameter and an average length of 35 mm, were used as fiber in the composite mix.

### 2.2. Mix Design and Cubic Conprssive Strength

The concrete mix design was prepared for obtaining a concrete target strength of grade 35 (35 MPa) with single mix design. The mix in the laboratory was selected specifically as 281.4 kg/m^3^ cement, 667.44 kg/m^3^ sand, 1001.16 kg/m^3^ gravel. The water cement ratio was 0.43. For the cementitious composite tube, the mix ratio was followed as Cement: river sand: quartz sand: water = 1.0:1.0:1.0:0.45. Cement: silica fume: steel fiber: superplasticizer ratio was 1.0:0.1:0.1:0.000992. The compressive strength determined from a 100 mm cube of core concrete and a cementitious composite tube achieved 23.42 MPa and 31.32 MPa at 7 days of curing, respectively.

### 2.3. Specimen Fabricaiton

The spiral hoop was placed inside the steel mold, and then the cementitious mixture was cast in three equal layers. The mold was placed on a vibrating table to achieve proper compaction. All specimens were covered by plastic bags after casting to confirm the intense hydration reaction during the first 4–6 h. The molds were removed after 24 h and then the tubes were cured in a water tank at approximately 26(±1) ℃ or for 3 days. The specimen was taken out of the water tank after 3 days of curing and dried for 2 h to achieve surface dry condition.

**Figure 3:**
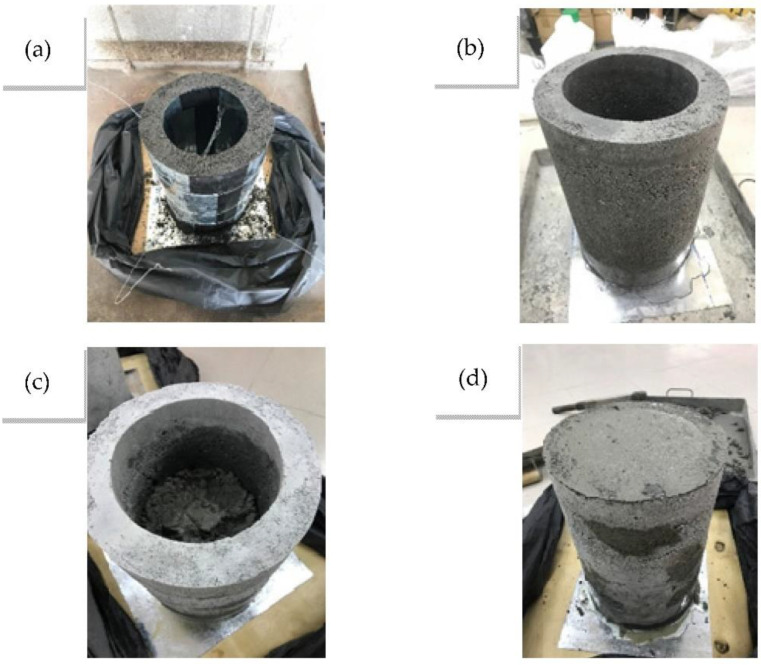
Tube fabrication procedure. (**a**) Tube pouring; (**b**) Surface drying; (**c**) Core concrete pouring; (**d**) Core capping.

Next, the freshly mixed concrete was poured into the cementitious tube in three equal layers and compacted with a 25 mm diameter rod, 30 times each layer. An additional concrete layer was cast over the top of every specimen to achieve a finished surface, and then sealed back into plastic bags for 24 h. All specimens were cured under water to finish the rest of the 28 days of hardening before being tested. Thus, the whole process period lasted 32 days. Fabrication process is depicted in Figure 3.

### 2.4. Hardened Property Test

All specimens were tested under axial compression with a universal testing machine. The specimens’ axial deformation was measured using two linear variable displacement transducers (LVDTs), marked as AS-1 and AS-2, of 100 mm gauge length attached to the middle region of the column. The axial strain for each specimen was calculated based on the average deformations recorded by the LVDTs. In addition, two 100 mm gauge length LVDTs were horizontally installed at mid-height to record the lateral deformations and subsequent strains. Both gauges were placed on two opposite sides of the surface. To record the axial load, a 100-ton load cell was placed at the top of specimen.

**Figure 4:**
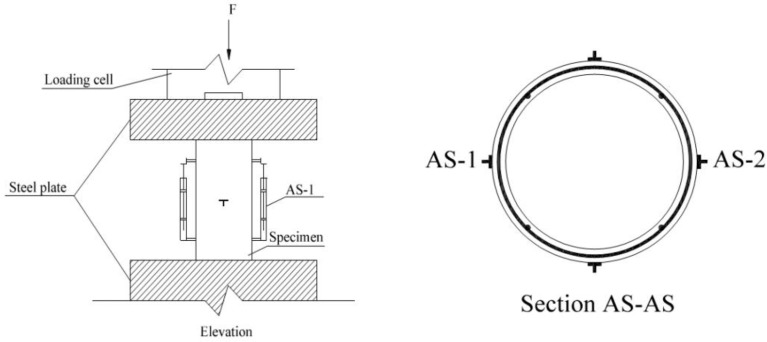
Test setup and instrumentation.

All of the LVDTs and load cell sensors were connected to a data logger before starting the test to collect the data. All columns were tested under a loading rate of approximately 1 kN/s, until they reached failure. Figure 4 shows the test setup and instrumentation followed in this experiment.

## 3. Results and Discussions

The test results were collected with data recording software, installed in a laptop that was connected to the data logger. The following sub-sections present and discuss the recorded results.

### 3.1. Load and Deflection Capacities

The ultimate load carrying capacity of unconfined specimens and cementitious composite tube-confined specimens is tabulated in Table 2. It can be seen that the load carrying capacity of tube-reinforced specimens was progressively improved. The enhanced loading capacity of tube-confined reinforcement specimens is a result of the external tube.

It directly provides the benefit of a higher load carrying capacity from cementitious composite tube confinement. As the independent variable of tube reinforcement, it can be said that the tube contributes significant lateral confinement to improve the load carrying performance. The increment in the ultimate load carrying capacity of CFPCCT specimens reached up to 40% higher than the unconfined specimens. Similar results were also presented for ferrocement-confined cylindrical concrete columns, where the same size specimens were tested [6]. It should be noted that the outer prefabricated tube carried both the axial load and lateral confining pressure, and thus contributed to an enhanced load carrying capacity.

The greater thickness of the prefabricated tube contributed to a higher load carrying capacity in this experiment. Moreover, it should be noted that the increment in load carrying is a nearly linear increase in this case. However, a tube with 35 mm thickness is already higher, and increases almost 50% of cross-sectional size compared to the diameter of the core concrete. Therefore, it is not recommended to provide higher thickness beyond this.

The axial deflection is related to the stiffness of the member. Therefore, axial deflection is reported in this study even though the column is very short. The ultimate axial deflection capacities of the test specimens are reported in Table 3. It is observed that the axial deflections, at both ultimate load and at failure, of group C-20-35 are higher than rest of the specimen groups. With the wall-thickness increasing, the axial deflections are 1.61, 1.58 and 1.29 times at the ultimate load deflection relative to group C-1, respectively. The higher axial deflection capacity considers that the ductile nature of cementitious composite tubes permits more lateral deformation from the composite materials and the hoop reinforcement.

### 3.2. Failure Modes and Cracking Behavior

**Figure 5:**
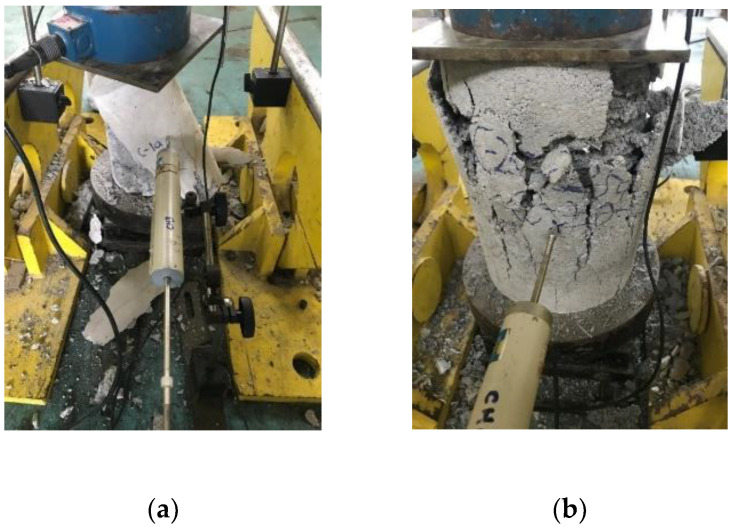

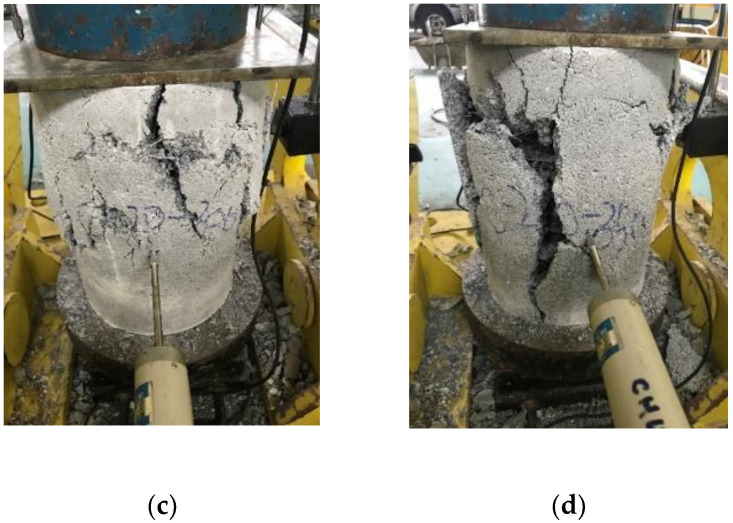
Typical failure mode of tested specimens. (**a**) C-1; (**b**) C-20-25; (**c**) C-20-30; (**d**) C-20-35.

The typical failure patterns for each group of tested specimens are presented in Figure 5. The failure of the cementitious composite tube-confined specimens, shown in Figure 5b–d, was similarly indicated by the formation of vertical cracks throughout the height of the entire outward surface; the width of the cracks increased with the increase of applied load, and ultimately failed. Bigger cracks appeared in all of the tube-confined specimens at the time of failure, which can be seen in Figure 5. The cracks in the cementitious tubes all had a width of more than 5 mm. However, during the failure of the non-reinforced specimens (Figure 5a) micro-cracks were observed in the specimens due to the application of load, and suddenly failed at the ultimate load. Furthermore, all of the non-reinforced specimen columns show a catastrophic collapse with the disintegration of structural integrity. The failure of all of the tube-confined specimens was due to the failure of the cementitious tube, where surface peeling in the tube was also observed.

For the CFPCCT specimens and mortar tube specimens, it is exhibited that cracking first occurred at the mid-height region, when the axial load reached about 80% of ultimate strength. Multiple cracks prominently extended following these initial cracks. The concrete cover peeled off at the same moment and the hoops were partially exposed at failure, as shown in Figure 5. For the control specimens, the first cracking point occurred throughout the wall surface when the axial load reached about 95% of ultimate value; then, a significant drop of loading was observed with an explosive failure of the specimens. From Figure 5, it can be seen that the 25 mm- and 35 mm-thick tube-confined specimens disintegrated upon failure. However, the 30 mm-thick tube-confined specimen showed a better performance in terms of disintegration. The disintegration of the specimens might be due to the fact that the tube also carried the axial load directly from the loading device. However, surprisingly, the spiral reinforcement was not yielded. This might be due to the fact that the reinforcement was not tied with any vertical reinforcement. In addition, the direct load applied to the tube was higher than that of the outward passive bursting pressure [6,7,8].

### 3.3. Stress-Strain Response

**Figure 6:**
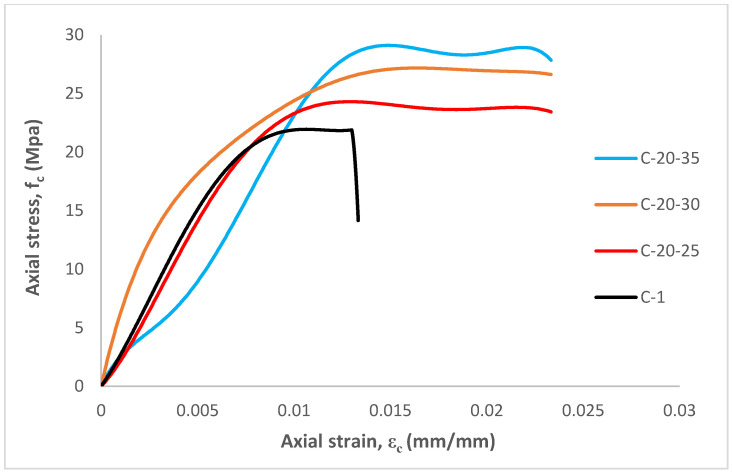
Stress-strain curves of specimens.

Figure 6 presents the stress-strain responses of the tested specimens. The stress-strain curves were established from thousands of original data points, obtained from the experimental test. Theoretically, the yield displacement is the yield point of an equivalent bilinear response curve that provides an equal area to that of the response curve [23]. In this study, the ultimate strain $\varepsilon_{c}$ is considered to be the axial strain at the failure point when the load drops 20% from the peak load [23]. However, the yield point of all specimens in this study refers to the first cracking load.

A descending branch, after the peak in the stress-strain curve, was expected for the tube confined specimens. However, the curve becomes flat to nearly flat after the peak strength and the compressive strength is reached, before the rupture of tube confinements. This behavior might be due to the fact that the external tube acted as an integrated part of the composite system, which carries both lateral and axial loads simultaneously. The stress-strain curves of the tube-confined specimens are quite similar. However, sudden failure was observed for unconfined concrete specimens. If the stress-strain curve terminates at a concrete stress higher than the compressive strength of non-reinforced specimens, the tube confinement is still working to enhance the strength [12,16]. It must be mentioned that the confining tube also carried the direct axial load, which contributes to the enhanced stress-strain responses.

It can be seen that the axial stress-strain curve of the C-20-30 specimens show the smoothest changing process, from load applying to failure. Conversely, the C-1 specimens show that there is a clear turning point, after the stress goes through the peak and drops sharply. This is also reflected in the testing procedure when there is an explosion occurred on C-1 specimens due to the limit of ductility.

The performance of the C-20-35 specimens is slightly different to the others around the yield point. Furthermore, they all pass through the linear stage before yield. In terms of its mechanical performance, the C-20-35 shows the higher compressive strength and better axial deformation, which means the higher wall-thickness of CFPCCT columns are functioning. However, it does not continue to 81.19% of ultimate compressive strength. In contrast to the high performance confined concrete tube, the wall-thickness of the cementitious composite tube is more sensitive to the load carrying capacity. Thus, it can be said that the loading carrying capacity will increase with an increasing wall-thickness of CFPCCT columns.

### 3.4. Ductility of Confinement

**Figure 7:**
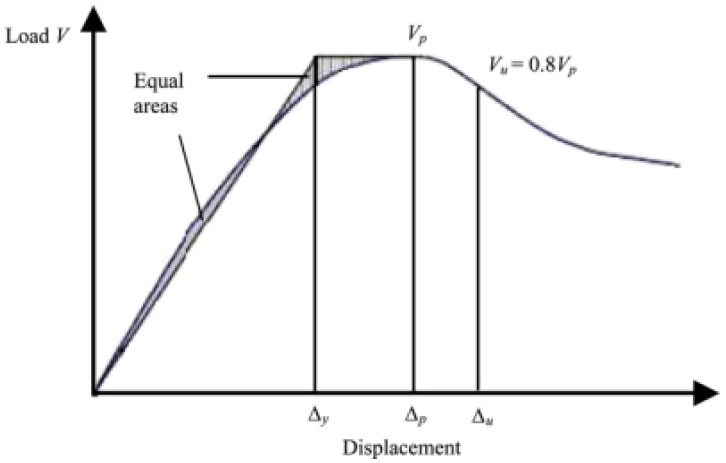
Definition of the ductility ratio.

The tendency of a structural member to deform under large strains without fracturing is referred to as the ductility of that member, which is a desirable property of any structural element that provides warning before failure. On the one hand, the ductility of any specimens tested under compression can be computed in terms of displacement ductility, a ratio of the axial displacements corresponding to the yield and 0.85 ultimate loads [6]. On the other hand, Wang et al. [24] state that the ductility ratio of axial displacements is from the yield to 0.8 ultimate loads, as shown in Figure 7. This study adopts yield to 0.8 ultimate due to the fact that Kaish et al. [6] proposed the method for ferrocement, which is a well-known cementitious composite.

The evaluated specimen’s ductility is presented in Table 4. However, specimens that failed before even reaching 0.8 of ultimate stress on the descending branch are judged as the failure point directly, rather than theoretically calculating to achieve the perfect model, against the reality of the C-1 groups. The result shows that all tube reinforced specimens have a better performance of ductility ratio than non-reinforced specimens. The ductility values of the specimens are compared with the unconfined specimens. For the 25 mm, 30 mm and 35 mm wall-thickness cementitious composite tube-confined specimens groups, the ductility ratios are 3.1971, 3.3768 and 3.4247, respectively. Therefore, the results lead to the conclusion that higher wall-thickness causes better performance on ductility.

## 4. Conclusions

In this investigative study, concrete-filled prefabricated cementitious composites tube (CFPCCT) columns with different tube wall-thickness were tested under axial compression. Based on the obtained test results, the following conclusions are drawn:

1. It is observed that the concrete-filled prefabricated cementitious composite tube columns performed a better load carrying and ductility capacity over unconfined concrete cylindrical specimens.
2. For the cementitious tube confined composite column specimens, the first crack occurred at the mid-height region when the axial load reached approximately 80% of the ultimate strength. After that, multiple cracks prominently extended with surface peeling. The 25 mm and 35 mm thick tubes showed complete disintegration upon failure. The cementitious composite tube with 30 mm wall thickness performed better in terms of failure behavior.
3. The strength and stress-strain behavior of CFPCCT columns enhanced with increased wall-thickness of the cementitious composite tube. This behavior is prominent as the external tube also directly carried the axial load.
4. Further investigation is recommended on this aspect to optimize the tube parameters for optimized performance of CFPCCT columns, together with suitable analytical models, to establish its practical application.

## Figures and Tables

**Table 1:** Tested specimens’ details.

Groups	Specimens	Wall Thicknesses (mm)	Hoop Spacing (mm)	Type of Confinements
C-20-25	C-20-25a	25	20	Cementitious composite tube confined
	C-20-25b	25	20	
C-20-30	C-20-30a	30	20	Cementitious composite tube confined
	C-20-30b	30	20	
C-20-35	C-20-35a	35	20	Cementitious composite tube confined
	C-20-35b	35	20	
C-1	C-1a	-	-	Unconfined
	C-1b	-	-	

**Table 2:** Tested specimens’ load carrying capacity.

Specimen	Yield Load (kN)			Ultimate Load (kN)			Ultimate Axial Stress (MPa)	Increment in Load to C-1 (%)
	Measured		Average	Measured		Average		
C-1	324.92	329.40	327.16	387.47	389.65	388.56	21.99	-
C-20-35	382.67	375.29	378.98	466.22	481.25	473.74	30.79	40.0
C-20-30	395.86	392.36	394.11	488.50	480.16	484.33	27.42	24.7
C-20-25	391.33	403.84	397.59	483.64	488.72	486.18	24.19	10.0

**Table 3:** Axial deflections of all specimens.

Specimen	Axial Deflection at Ultimate Load (mm)			Axial Deflection at Failure (mm)			Relative to C-1 in at Ultimate Load
	Measured		Average	Measured		Average	
C-1	3.0	3.1	3.1	3.1	3.1	3.1	-
C-20-25	3.9	4.1	4.0	6.3	6.4	6.4	1.29
C-20-30	4.7	5.1	4.9	6.9	7.0	7.0	1.58
C-20-35	4.9	5.1	5.0	7.4	7.6	7.5	1.61

**Table 4:** Ductility of the tested specimens.

Specimen	Strain at Yield Stress (mm/mm)	Strain at 0.8 of Ultimate Stress (mm/mm)	Ductility Ratio
	Average	Calculated	
C-1	0.0052	0.0103	1.98
C-20-25	0.0067	0.0213	3.1791
C-20-30	0.0069	0.0233	3.3768
C-20-35	0.0073	0.0250	3.4247

## Data Availability

Not applicable.
